# Projecting Global Occurrence of *Cryptococcus gattii*

**DOI:** 10.3201/eid1601.090369

**Published:** 2010-01

**Authors:** Deborah J. Springer, Vishnu Chaturvedi

**Affiliations:** New York State Department of Health, Albany, New York, USA; University at Albany School of Public Health, Albany

**Keywords:** Fungal pathogen, Cryptococcus gattii, yeast, fungus, environmental niche, geographic distribution, synopsis

## Abstract

*Cryptococcus gattii* and *C. neoformans* cause pulmonary and systemic cryptococcosis. Recently, *C. gattii* was recognized as a distinct pathogen of humans and animals. We analyzed information from 400 publications (1948–2008) to examine whether the fungus occurs globally. Known distribution of *C. gattii* is possibly limited because specialized reagents for differentiation from *C. neoformans* are not readily available and not always used, and environmental surveys are patchy. However, autochthonous reports of *C. gattii* cryptococcosis have now been recognized from tropical and temperate regions. An ongoing outbreak in western Canada strengthens the case that the range of the pathogen has expanded. A few studies have highlighted differences in cryptococcosis between *C. gattii* and *C. neoformans*. More than 50 tree species have yielded *C. gattii* especially from decayed hollows suggesting a possible ecologic niche. This pathogen merits more attention so its environmental occurrence and role in cryptococcosis can be accurately determined.

## Abstract 2

This pathogen likely has wider distribution than is currently recognized.

The yeast genus *Cryptococcus* has been recognized for >125 years, first from fruit juice, milk, humans, soil, and pigeon droppings and from roosting areas (*1*). Although *C. neoformans* human infections were reported early in the 1900s, the overall number of cryptococcosis cases was extremely low. Cryptococcosis cases increased in Africa during 1947–1968, presumedly in association with the emergence of AIDS in the Congo River basin (*2*); however, no independent confirmation or laboratory data are available for this hypothesis. A unique variant, *C.* *neoformans* var. *gattii*, manifested by the unusual presence of elongated and cigar-shaped yeast morphology in cerebrospinal fluid, was first described in a Congolese Bantu boy (*3*,*4*).

Evans described and differentiated *C. neoformans* into 3 serologic types (A, B, and C) by agglutination (*5*). Diagnosis of cryptococcosis progressed further with identification of *C. neoformans* antibodies in body fluids and development of a latex agglutination test (*1*). Staib (*6*) developed a *Guizotia abyssinica* (Nigerseed) creatinine agar medium to distinguish pigment-producing *C. neoformans* from other *Cryptococcus* spp., which facilitated rapid screening of clinical and environmental samples for pathogenic *C. neoformans* isolates. A major advance in the classification and taxonomy of *C. neoformans* occurred with the discovery of a heterothallic, bipolar mating involved in the production of the perfect state for *C. neoformans* var. *gattii* (serotypes B and C). It was termed *Filobasidiella bacillispora* and differentiated from *F. neoformans* by production of smooth, elongate cylinder- to rod-shaped basidiospores (*7*).

Currently, *C. neoformans* is recognized as a species complex comprising *C. neoformans* var. *grubii* (serotype A) and *C. neoformans* var. *neoformans* (serotype D), which have distinct clinical manifestations and biological characteristics (*1*,*8*). *C. gattii* (serotypes B and D) was recognized as a species distinct from *C. neoformans* because of differences in basidiospore morphology, environmental niches, morphologic features in vivo, limited molecular identity (55%–61% relatedness of DNA), multiple gene genealogies, unique random amplified polymorphic DNA typing patterns, and inefficient cross-species mating with the production of sterile progeny and no recombination (*9*). During the previous 2 decades, the increased pace of discovery produced a new appreciation of the 2 major pathogenic species, namely, *C. neoformans* and *C. gattii*. This study aimed to critically examine published information about associated tree species, ecology, and geographic occurrence of *C. gattii* to infer its environmental distribution.

## Methods

We comprehensively searched for published reports using the PubMed database (US National Library of Medicine, National Institutes of Health) for 1948–2008. The keywords used in the search were *Cryptococcus* alone or in combination with *Cryptococcus gattii*; *Cryptococcus* *neoformans*; *Cryptococcus neoformans* var. *neoformans*; *Cryptococcus neoformans* var. *grubii*; *Cryptococcus neoformans* serotype A, B, C, D, or AD; and cryptococcosis alone or in combination with human, pigeon, and animal. Additionally, we scrutinized reference lists in publications obtained from PubMed searches for citations that had not been captured with our choice of keywords in PubMed searches. These citations were easily obtained by repeating the search criteria in the Web of Science (Thompson Reuters) and Google Scholar.

One of us (D.J.S.) independently examined the title, abstract, methods, data tables and figures of publications identified in the literature search. Information about *Cryptococcus* isolates, serotype, mating type, molecular type, geographic location, and other relevant details were entered into a master spreadsheet. All publications with adequate documentation of *C. gattii* by >1 valid laboratory methods were regarded as acceptable for inclusion.

## Results

From 400 potentially useful publications, we shortlisted ≈200 and identified 105 that provided information about primary isolations of *C. gattii* from clinical, veterinary, and environmental sources. Geographically, the reports originated from a total of 48 countries, although most reports concentrated on few areas (Table 1). Because a certain level of selection bias existed in this search process, we might have missed some relevant publications (*10*).

**Table 1:** Number of publications per geographic region reporting isolation of *Cryptococcus gattii*

Region	Environmental isolation	Clinical and veterinary isolation	Total no. reports
South America	18	12	30
Australia/New Zealand	8	16	24
North America	5	14	19
South-central Asia	9	9	18
Africa	2	12	14
Europe	3	12	15
Eastern Asia	0	6	6
Central America	1	5	6
Southeast Asia	0	3	3

### Distinguishing Features of *C. gattii*

*C. gattii* was easily and reliably differentiated from *C. neoformans* on creatinine dextrose bromthymol blue (CDB) medium. This work built on the discovery that *C. neoformans* can assimilate creatinine as sole source of carbon and nitrogen. Further modification in CDB medium led to development of canavanine-glycine-bromthymol blue agar, which has since become the differential medium of choice (*1*,*11*,*12*). Unfortunately, the medium is still not widely used in diagnostic laboratories, most likely because of limited availability from commercial suppliers.

*C. gattii* populations can be distinguished by the pairing of unknown isolates with compatible tester strains to distinguish *MATα* from *MAT****a*** strains. *MATα* is most prevalent clinically and environmentally, and *MAT****a*** is recovered less frequently (*7*,*13*,*14*). Four distinct *C. gattii* molecular subtypes (VGI, VGII, VGIII, and VGIV) have been recognized by PCR amplification of genomic DNA by using bacteriophage M13 single-stranded primers. Genotypes VGI and VGII are prevalent worldwide, and VGIII and VGIV are less common (Figure). Serotypes B and C are randomly dispersed among the M13 molecular types. Further molecular subtypes are now known to exist within the 4 molecular types (*15*).

**Figure:**
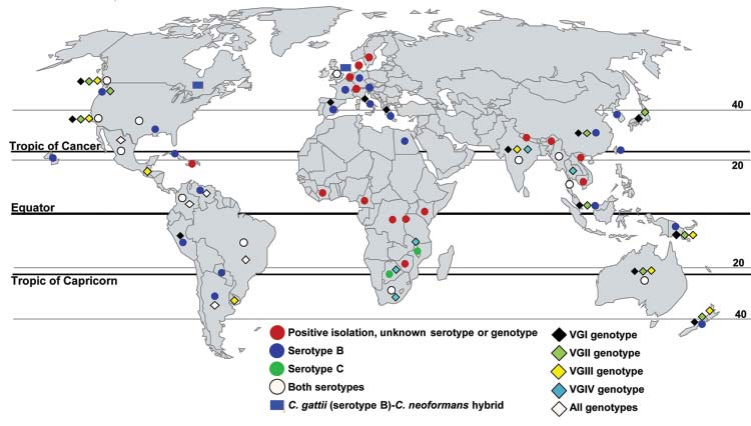
Worldwide isolations of *Cryptococcus gattii* from human clinical, veterinary, and environmental sources. Circles indicate serotype information, diamonds indicate genotype information, and rectangles indicate hybrids between *C. gattii* and *C. neoformans*. Existing reports and survey are patchy, and several areas between positive regions share tree species (Table 2) and climatic conditions and would most likely harbor the pathogen. Thus, *C. gattii* is likely to be more widely distributed than documented.

The VGI molecular genotype has been reported from many areas and from 2 *C. neoformans–C. gattii* hybrids reported from Canada and the Netherlands (*14*,*16*). VGII has been reported from the Western Hemisphere, Australasia, Asia, and Africa and is reportedly the most fertile and virulent of the strains responsible for infection in healthy and immunocompromised humans and animals. This strain also is associated with an ongoing outbreak of *C. gattii* cryptococcosis from Vancouver Island, British Columbia, Canada (*14*). VGIII, which contains both serotypes B and C, is most commonly reported from South America but also is reported from North America, Central America, Australasia, and Southern Asia. VGIV, frequently associated with serotype C, has been reported from Africa and South and Central America. Further experimental studies on VG genotypes are needed to explain possible connections between distribution of various genotypes and propensity to cause cryptococcosis among exposed hosts.

### *C. gattii* in Clinical and Environmental Specimens

Initially, reports of *C. gattii* originated from human clinical samples in tropical and subtropical regions, including portions of Africa, Europe, Australia, the United States, and South America. This accounted for the long-held impression that *C. gattii* is a tropical or subtropical pathogen (*4*,*17*,*18*). More recent clinical isolations from temperate regions in the United States, Canada, Europe, and Asia have greatly expanded the incidence areas of *C. gattii* (*4*). Accordingly, *C. gattii* has been reported from such diverse countries as Argentina, Austria, Canada, China, Congo, India, Italy, Japan, South Korea, the Netherlands, Spain, South Africa, United Kingdom, United States, and Democratic Republic of Congo, and this list expands every year. Infections in domestic animals, such as goats, dogs, cats, and horses, are common in Australia, New Zealand, Canada, and Brazil (*4*,*19*,*20*). Additionally, infections are reported in migratory, water-dwelling animals, including porpoises and dolphins (*21*). *C. gattii* also was associated with native animals in Australia (koala, echidna), New Zealand (kiwi), Africa (cheetah), and Canada (squirrel) (*14*,*19*,*22*). Non-native zoo animals (koala, ferret, tapir, cheetah, llama) and exotic birds (cockatoo and parrots) have been affected with cryptococcosis caused by *C. gattii* in Australia, Canada, the United States, and Cuba (*23*). Thus, *C. gattii* affects a wide variety of native and domestic animals in regions of known clinical presence. Because, in comparison with human clinical samples, veterinary samples are less frequently analyzed for and diagnosed with *C. gattii* infection and subsequently reported, we suggest that the actual infection rates of *C. gattii* in animal populations are possibly much higher than presently known.

Published literature on the environmental isolation of *C. gattii* is patchy, sporadic, and centered in geographic regions reporting a high clinical incidence of *C. gattii* cryptococcosis. This is true for Canada, South America, and Australia. However, in India the environmental prevalence of *C. gattii* appears more pervasive than the reported prevalence of the fungus in clinical specimens (Figure). The first environmental isolation of *C. gattii* (serotype B) was reported by Ellis and Pfeiffer from *Eucalyptus* trees in 1990 (*24*) after unsuccessful attempts at environmental isolations from the same tree species in Oklahoma (*18*) and California (*25*). The first environmental isolation of serotype C was reported in 1998 from almond trees in Colombia (*26*). Environmental sampling is much more limited than clinical sampling because clinical isolates are a public health priority.

*C. gattii* serotype B is the most prevalent serotype in clinical and environmental samples (*17*,*18*). Curiously, *C. gattii* serotype C is a less common constituent of clinical and environmental isolations even though it is associated with AIDS patients and immunocompetent persons (*27*). *C. gattii* serotype C has been isolated from humans in clinical samples from India, the Western Hemisphere, and Africa (*18*,*27*). Although serotype C is rarely isolated from the environment, its most notable isolation occurred in association with detritus around nonnative almond trees in Colombia (*26*). More recent and extensive clinical surveys combining serotyping and molecular typing suggest that serotype C is less rare or restricted than previously thought (*27*). Future studies are unlikely to provide serotype information because the commercial typing reagents are no longer readily available, and thus genotypes will be the primary means to correlate strain characteristics with their environmental and clinical prevalence.

Existing reports and surveys of *C. gattii* from human clinical, veterinary, and environmental sources are patchy (Figure). Several areas between positive regions would most likely harbor the pathogen. Thus, *C. gattii* is likely to have a wider geographic distribution than documented. The environment has not been systematically explored to identify the source of *C. gattii* in the Congo River basin, where the first definitive report of *C. gattii* emerged. Such environmental surveys are imperative in view of reports of *C. gattii* cryptococcosis from a number of African countries (*3*,*17*,*27*,*28*).

### *C. gattii* and Trees

Ellis and Pfeifer reported the first environmental isolation of *C. gattii* in 1990 in Australia from wood, bark, leaves, and plant debris of *Eucalyptus* trees (*24*). Although *Eucalyptus* is present in many of the areas known to have *C. gattii* cryptococcosis, the actual isolation of *C. gattii* from *Eucalyptus* trees is rare outside Australia, despite extensive sampling. Imported *Eucalyptus* has not been associated with the environmental presence of *C. gattii* in Spain, central Africa, or Canada, and most *Eucalyptus* trees tested in Papua New Guinea, Egypt, and Italy were negative for *C. gattii*. Furthermore, early environmental surveys for *C. gattii* in imported *Eucalyptus* spp. rarely included other local tree species for testing (*4*). Although understandable, this was unfortunate because *C. gattii* is now known to have extensive associations with other tree species.

Evidently, *C. gattii* is established ecologically in trees other than *Eucalyptus* in many parts of the world, as supported by *C. gattii* association with native trees in Canada, Brazil, Colombia, India, and Argentina (Figure). *C. gattii* has been reported from 54 tree species; most (77%) are angiosperms; gymnosperms account for 23% of positive species (Table 2). Gymnosperms and angiosperms can develop decayed hollows, which differ in biochemical composition, available nutrients, presence of water, microbial communities, and fungal associations (*29*). *C. gattii* exhibits associations with the gymnosperms *Abies* spp., *Arbutus menziesii* var. *menziesii, Cedrus* spp., *Abies grandis*, *Picea* spp., *Pinus* spp., *Pseudotsuga menziesii* var. *menziesii*, and *Thuja plicata* in Canada; *Pinus radiate* (Monterey pine) and *Cupressus lusitanica* in Colombia; and *Cedrus deodara* and *Cupressus sempervirens* in Argentina. Angiosperms other than *Eucalyptus* spp. have been reported positive for *C. gattii* from North America, South America, Africa, and India. Like *Eucalyptus* spp., other angiosperm tree species reported as hosts for *C. gattii* have been extensively exported from their native areas (Table 2). Two prominent examples are *Ficus* spp. and *Terminalia* spp. (almond) trees. *Ficus* spp. are widely distributed in the tropics and subtropics, and many are exported as ornamentals. *Ficus* spp. have been recorded as *C. gattii* hosts in Brazil and Colombia but not in other regions (*30*).

**Table 2:** Tree species recorded as testing positive for *Cryptococcus gattii*

Location	Species (common name)	Native and exported tree ranges
Argentina	*Acacia visco* (arca), Cedrus deodara* (deodar cedar), Cupressus sempervirens* (Mediterranean cypress), *Eucalyptus microcorys* (tallowwood), *Tipuana tipu* (rosewood), *Ulmus campestris* (English elm)	Australia, Africa, Asia, Britain, Canada, Central America, England, Europe, Japan, South America, United States
Australia	*Angophora costata* (smooth bark apple*), E. blakelyi* (Blakely's red gum), *E. camaldulensis* (red river gum), *E. gomphocephala* (tuart tree), *E. grandis* (rose gum*), E. microcorys* (tallowwood), *E. rudis* (flood gum*), Eucalyptus* spp., *E. tereticornis* (forest red gum), *E. tetrodonta* (Darwin stringybark), *Syncarpia glomulifera* (turpentine)	Australia, Africa, Asia, Caribbean, Hawaii, Indonesia, New Zealand, Papua New Guinea, United States, South America, US Virgin Islands, British Virgin Islands
Brazil	*Adenanthera pavonina* (circassian seed), *Cassia grandis* (carao), *Erythrinia velutina* (coral tree), *E. camaldulensis* (red river gum), *E. microcorys* (tallowwood), *Eucalyptus* spp., *Ficus* spp., *Guettarda acrena, Moquilea tomentosa* (pottery tree)	Australia, Africa, Asia, Caribbean, Central America, Fiji, New Zealand, South America, United States, US Virgin Islands, British Virgin Islands
Canada	Abies grandis* (grand fir), *Abies* spp.* (fir), *Acer* spp.(maple), *Alnus rubra* (red alder), *Aluns* spp. (alder), Arbutus menziesii* (Pacific madrone*), Cedrus* spp.* (cedar), *Picea* spp.* (spruce), *Pinus* spp.* (pine), *Prunus emarginata* (bitter cherry), Pseudotsuga menziesii* (coastal Douglas fir), *Quercus garryana* (Garry oak), Thuja plicata* (western red cedar)	Australia, Europe, New Zealand, North America, South America
Colombia	*Acacia decurrens* (black wattle), Coussapoa sp, Croton bogotanus, C. funckians (C. gossypiifolius), Cupressus lusitanica* (Mexican cypress), *E. camaldulensis* (red river gum), *E. globulus* (Tasmanian blue gum), *Ficus soatensis* (rubber Savanna), Pinus radiata* (Monterey pine), *Terminalia catappa* (almond)	Africa, Asia, Australia, British Isles, Canada, Caribbean, Costa Rica, Europe, Costa Rica, Hawaii, Indonesia, Mediterranean region, Mexico, New Zealand, Pacific Islands, Papua New Guinea, Japan, United States, South America, US Virgin Islands, British Virgin Islands
India	*Acacia nilotica* (thorn tree), *Azadirachta indica* (neem tree), *Cassia fistula* (golden shower tree), *Cassia marginata, E. camaldulensis* (red river gum), *E. citriodora* (lemon-scented gum), *Eucalyptus* spp., *Mangifera indica* (mango), *Manilkara hexandra* (margosa), *Mimusops elengi* (bullet wood or Indian madlar tree), *Pithecolobium dulce* (Manila tamarind), *Polyalthia* longifolia (Indian mast tree), *Syzygium cumini* (java plum), *Tamarindus indica* (tamarind), *Terminalia arjuna* (arjuna)	Africa, Asia, Australia, Caribbean, Central America, Hawaii, Indonesia, Malaysia, Pacific Islands, Philippines, Portugal, South. America, New Zealand, United States, US Virgin Islands, British Virgin Islands
Egypt, Italy, Mexico, United States	*E. camaldulensis* (red river gum)	Africa, Australia, Caribbean, New Zealand, United States, South America, South Asia, US Virgin Islands, British Virgin Islands

*Gymnosperm tree species.

### *C. gattii* vis-à-vis *C. neoformans*

Cryptococcosis due to *C. gattii* is unlikely to be recognized in the laboratory without heightened awareness and sustained effort to differentiate these 2 closely related pathogens. Given the much more recent recognition of *C. gattii*, historical reports are likely to mention only *C. neoformans*; this is a major consideration in evaluating historical publications on cryptococcosis due to *C. gattii*. Pigeon droppings are a known ecologic niche for *C. neoformans* because the pathogen is predominantly isolated from avian environments or areas contaminated with avian feces (*1*). Thus, urban dwellings frequented by pigeons and containing accumulated pigeon droppings are an important reservoir for human and animal infections.

Pigeons are not known to acquire symptomatic disease but can carry yeast on feathers, skin, crops, or cloaca (*1*). Other animals reported positive for *C. neoformans* include macaw, swan, parakeet, Guenon monkey, fox, potoroo, and sheep (*1*). Thus, exotic and migratory birds and domestic and wild animals can be carriers or susceptible hosts for *C. neoformans*. The overwhelming association with avian droppings and environment, especially pigeons, sets *C. neoformans* apart from *C. gattii*. The ecologic niches for *C. neoformans* and *C. gattii* appear to be distinct.

Few reports exist of isolations of *C. neoformans* and *C. gattii* from the same habitats with the recognitions of natural hybrids between the 2 species. For instance, *C. neoformans* and *C. gattii* have been isolated from same sources, such as *Eucalyptus* spp. or *Syzygium cumini* trees or bird feces (*1*,*30*,*31*). *C. grubii* association with trees might represent fecal contamination by birds inhabiting these trees. Hybrid strains have been isolated from samples of bird feces in urban areas of South America and from patient samples obtained from the Netherlands and Canada (*16*). The existence of these hybrid strains suggests that at least in some parts of the world *C. neoformans* and *C. gattii* occupy either the same ecologic niche or closely overlapping areas.

## Discussion

We suggest that *C. gattii* is an environmental pathogen with a specialized ecologic niche on the basis of accumulated reports of its widespread isolation from domestic and native animals, clinical presence in temperate climatic regions, increasing reports of isolations from native trees in temperate regions, and recapitulation of life cycle in association with plant material. The characteristics of such environmental pathogens include absence of any recognized animal host and maintenance of virulent traits by specific environmental associations. This concept has been well developed for a number of other environmental pathogens, such as *Mycobacterium ulcerans* (*32*) and *Burkholderia* spp. (*33*). The 54 tree species recorded positive for *C. gattii* are native to tropical, subtropical, and temperate regions of the world. Additionally, many of these trees are more widely distributed than their documented native range(s) indicate because of extensive exportation and cultivation, which suggest further expansion of the known range of *C. gattii*.

A corollary of this environmental distribution of the fungus is the diagnosis of autochthonous *C. gattii* cryptococcosis in native and domestic animals in Europe, Africa, Australia and New Zealand, and the Western Hemisphere, suggesting that habitats of many of these animals overlap ecologic niches with the fungus (*19*). A consistent feature of the association of *C. gattii* with trees is isolation of the fungus from decayed hollows of angiosperm and gymnosperm species (*24*,*30*). Decayed wood hollows develop slowly and are distinct ecologic niches inhabited by specialized microbial communities (*29*). Microbes that use wood or decayed hollows require specialized adaptations to inhabit this ecologic niche, which also offers a refuge from deleterious biotic and abiotic factors. Decayed hollows are characteristics of mature trees and thus occur most frequently in forested regions or rural to semirural areas with mature trees (*29*). This pattern is consistent with recognition of *C. gattii* cryptococcosis in Canada, Australia, Africa, Asia, and parts of South America. In some instances, especially in temperate areas, *C. gattii* has been isolated from trees in parks, on college campuses, and in zoos and animal refuges (*24*,*31*). Recent studies provide additional evidence for this specialized ecologic niche in trees and tree hollows by documenting long-term associations of *C. gattii* with trees, including seasonal variations in its isolation, and genetic recombination indicative of sexual and/or asexual mating in association with trees and tree hollows (*13*). An experimental study has recapitulated the sexual life cycle of the fungus in the laboratory on *Arabidopsis* *thaliana* and *Eucalyptus* spp. seedlings with production of easily airborne sexual spores (basidiospores) thus supporting the universal dispersal hypothesis, which suggests that most of the free-living microbial eukaryotes are likely to be globally distributed (*34*).

The association of *C. gattii* with woody materials distinguishes this species from *C. neoformans* niche in soil and pigeon droppings. Several publications provide additional evidence for this inference: 1) Escandon demonstrated that *C. gattii* can survive in live almond trees and can contaminate the soil in immediate surrounding (*35*); 2) mating has been associated with live plants and wood (*13*,*36*); and 3) positive soil sample are mostly isolated near positive trees and have been contaminated with woody plant debris.

*C. gattii* potentially can be dispersed through export of trees and woody products, air currents, water currents, and biotic sources, such as birds, animals, and insects. The ability of *C. gattii* to associate with vesicular elements in wood blocks, to survive in the vasculature of live almond trees, and to spread into soil (*35*,*37*) suggests that the pathogen can spread through the exportation of wood and trees (*24*). Historically, *Eucalyptus* trees have been implicated in the spread of *C. gattii* to different areas in the world (*24*). Recently, *Pinus radiate*, *Cedrus deodara*, *Cupressus sempervirens*, *Cupressus lusitanica*, and *Terminalia catappa* (almond) have been recognized as *C. gattii* hosts; these trees have been widely exported from their native ranges as ornamental or commercially valuable trees. The evidence for *C. gattii* dispersal by wind and air currents is limited, but fungal isolations from air samples have been obtained around positive trees in Canada and India.

The following observations suggested dispersal of *C. gattii* in water or water currents: 1) naturally infected porpoises and dolphins have been identified, 2) the fungus has been isolated from natural freshwater and saltwater samples in British Columbia and from contaminated water in habitats of captive animals, and 3) *C. gattii* can survive in water in vitro for long periods (*21*). Multiple reports have suggested that birds and animals could play a role in dispersal of *C. gattii* to geographic areas presently uninhabited by *C. gattii*. Isolation of the fungus from psittacine bird excrement in South America is suggestive because many of these birds fly long distances and are migratory or are exported as exotic pets or exhibit items for zoos (*31*). Other native animals that could help in *C. gattii* spread include koalas in Australia (*22*), squirrels and porpoises in the Pacific Northwest (*19*), and dolphins (*21*) in North America.

*C. gattii* is likely to be acquired in areas where mature trees are abundant either in forested or rural to semiurban settings. We derived this conclusion from published clinical reports on *C. gattii* cryptococcosis in Aborigines in Australia, native Africans in the Congo River basin, Canadians who visited parks and forests on Vancouver Island, and a Spanish farmer and Italian farmer (*38*,*39*). A common theme among these clinical cases is presence of and human exposure to mature trees. Recovery of identical *C. gattii* strains from environmental sources from Canada and human clinical specimens from Italy strongly suggest that the point source of infection is the immediate vicinity of patients’ residences (*14*,*39*). Association of *C. gattii* with decayed woody hollows, bark, and tree debris also suggests a role for mature trees (*19*,*24*).

Infections reported in domestic and wild animals in Australia, New Zealand, Africa, Spain, the United States, and Canada provide another important clue to risk areas for *C. gattii* acquisition (*19*). Overwhelmingly, these infections are reported from animals that either reside in or are exposed to areas with an abundance of mature trees. This situation is somewhat analogous to the fungus *Blastomyces dermatitidis*, another elusive primary pathogen, which causes blastomycosis. Some similarities in characteristics include clustered infection patterns in humans and mammals; increases in exposure risk from outdoor activities, and restricted and infrequent environmental isolations (*40*).

## Conclusions

*C. gattii* is a globally established primary fungal pathogen with a specialized ecologic niche on trees and in hollows of trees. Future epidemiologic studies and environmental surveys are likely to reveal the extent of *C. gattii* prevalence in different environments especially in areas with known incidence of cryptococcosis but no reported isolations of *C. gattii*. Such information will be helpful in devising strategies to manage potential outbreaks of cryptococcosis. More clinical studies are also needed to follow up the course and outcome of *C. gattii* cryptococcosis, the salient point by which this fungus can be differentiated from the disease caused by *C. neoformans*, and any changes in patient management strategies.
